# Epidemiological Observations on the Association Between Anosmia and COVID-19 Infection: Analysis of Data From a Self-Assessment Web Application

**DOI:** 10.2196/19855

**Published:** 2020-06-11

**Authors:** Fabrice Denis, Simon Galmiche, Aurélien Dinh, Arnaud Fontanet, Arnaud Scherpereel, Francois Benezit, François-Xavier Lescure

**Affiliations:** 1 Inter-regional Cancer Institut Jean Bernard Le Mans France; 2 Emerging Diseases Epidemiology Unit Institut Pasteur Paris France; 3 Service de maladies infectieuses et tropicales Hôpital Raymond Poincaré Assistance Publique - Hôpitaux de Paris Garches France; 4 Service de pneumologie Centre Hospitalier Régional Universitaire de Lille Lille France; 5 Service de maladies infectieuses et réanimation médicale Centre Hospitalier Universitaire de Rennes Pointchaillou Rennes France; 6 Infectious and Tropical Diseases Department Bichat-Claude Bernard University Hospital and University of Paris Assistance Publique - Hôpitaux de Paris Paris France; 7 Team DesCID Infection, Antimicrobials, Modelling, Evolution - U1137 French Institute for Health and Medical Research, Institut national de la santé et de la recherche médicale Paris France

**Keywords:** COVID-19, anosmia, epidemiological surveillance, self-assessment, web application, outbreak, symptoms, self-assessment, surveillance, epidemiology

## Abstract

**Background:**

We developed a self-assessment and participatory surveillance web application for coronavirus disease (COVID-19), which was launched in France in March 2020.

**Objective:**

Our objective was to determine if self-reported symptoms could help monitor the dynamics of the COVID-19 outbreak in France.

**Methods:**

Users were asked questions about underlying conditions, sociodemographic status, zip code, and COVID-19 symptoms. Depending on the symptoms reported and the presence of coexisting disorders, users were told to either stay at home, contact a general practitioner (GP), or call an emergency phone number. Data regarding COVID-19–related hospitalizations were retrieved from the Ministry of Health.

**Results:**

As of March 29, 2020, the application was opened 4,126,789 times; 3,799,535 electronic questionnaires were filled out; and 2,477,174 users had at least one symptom. In total, 34.8% (n=1,322,361) reported no symptoms. The remaining users were directed to self-monitoring (n=858,878, 22.6%), GP visit or teleconsultation (n=1,033,922, 27.2%), or an emergency phone call (n=584,374, 15.4%). Emergency warning signs were reported by 39.1% of participants with anosmia, a loss of the sense of smell (n=127,586) versus 22.7% of participants without anosmia (n=1,597,289). Anosmia and fever and/or cough were correlated with hospitalizations for COVID-19 (Spearman correlation coefficients=0.87 and 0.82, respectively; *P*<.001 for both).

**Conclusions:**

This study suggests that anosmia may be strongly associated with COVID-19 and its severity. Despite a lack of medical assessment and virological confirmation, self-checking application data could be a relevant tool to monitor outbreak trends.

**Trial Registration:**

ClinicalTrials.gov NCT04331171; https://clinicaltrials.gov/ct2/show/NCT04331171

## Introduction

Web-based self-reporting of symptoms is a growing field and has been used to improve survival in oncology [1,2]; it can be used as a participatory surveillance tool for coronavirus disease (COVID-19) or other influenza-like illnesses as well [3,4]. We thought of applying the same technology to optimize patient triage for COVID-19 patients in France and alleviate the burden on emergency call centers. A self-assessment and participatory surveillance website [5] was developed and launched during the growing phase of the COVID-19 epidemic in France in March 2020. Our objective was to determine if self-reported symptoms could help monitor outbreak dynamics in France. We report here the analysis of the first 13 days of web application usage.

## Methods

Users were recruited via national media campaigns in France, including social media, radio, and magazine campaigns, between March 17-29, 2020. Participants were recruited through the maladiecoronavirus.fr website [5]. Respondents provided information on sociodemographic data, zip code, and coexisting disorders anonymously. They were asked about nine symptoms associated with possible COVID-19 infection—fever (body temperature >37.7°C), unusual cough, shortness of breath, sore throat, muscle aches, diarrhea, anorexia, and asthenia. Anosmia, a loss of the sense of smell, was added on March 21, 2020. Following symptom reporting, a notification was sent, recommending the user either to stay at home and use the website again in case of evolving symptomatology (self-monitoring), or to contact a general practitioner (GP), or to call an emergency number if they reported dyspnea or anorexia. A questionnaire was built according to Chinese reports and French experience [6]. The website was not considered a medical device by regulatory authorities since no tracking was performed and data were anonymous. We compared the distribution of web-based, self-reported symptoms to that of hospitalized COVID-19 patients according to Ministry of Health reports. Spearman correlation coefficients were used for statistical analysis.

## Results

**Figure 1:**
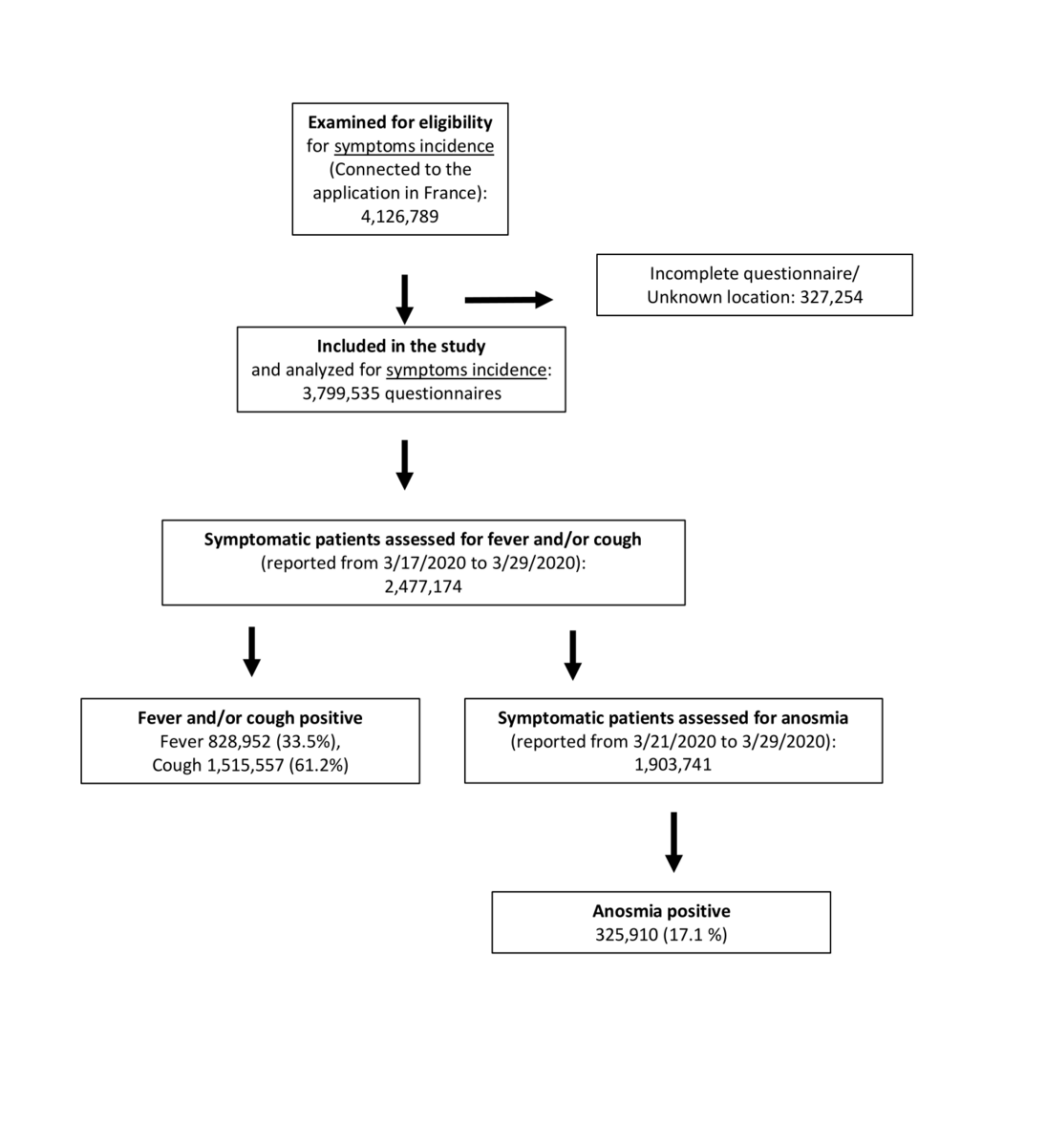
Flowchart of the study population.

Between March 17-29, 2020, the website was accessed 4,126,789 times; 3,799,535 electronic questionnaires were filled out; and 2,477,174 users had at least one out of the nine symptoms included in the questionnaire (Figure 1). In total, 1,322,361 (34.8%) participants reported no symptoms. The remaining patients (median age 37 years; range 15-99 years) were directed to self-monitoring (858,878, 22.6%), GP visit or teleconsultation (1,033,922, 27.2%), or an emergency phone call (584,374, 15.4%).

**Figure 2:**
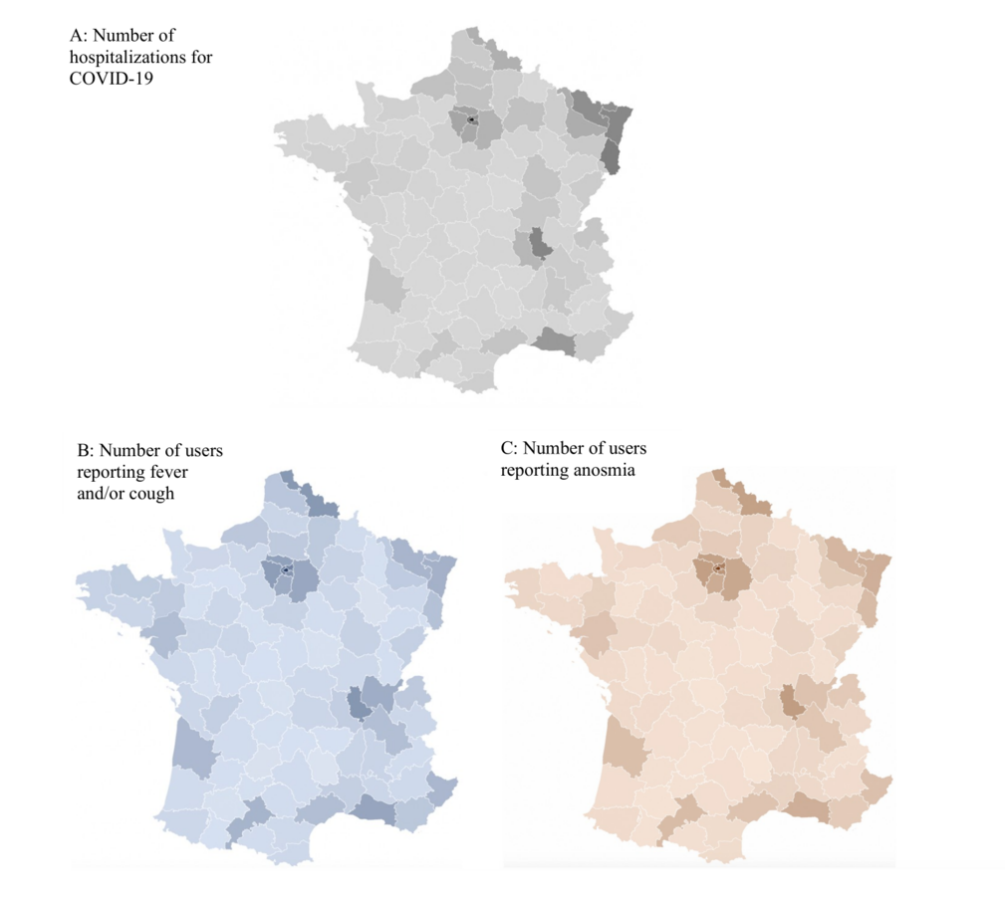
Maps displaying the correlation between fever and/or cough and anosmia with hospitalizations for COVID-19. (A) The cumulative number of hospitalized COVID-19–positive patients in France on March 29, 2020. (B) Fever and/or cough reported by users via the application; cumulative amount in French counties from March 17-29, 2020: 3,799,535 respondents (828,952 with fever and 1,515,557 with cough). (C) Anosmia reported by users; cumulative number from March 21-29, 2020: 325,910 positive respondents.

Of all symptomatic patients, anosmia was reported by 17.1% (325,910/1,903,741), fever was reported by 33.5% (828,952/2,477,174), and cough by 61.2% (1,515,557/2,477,174). Emergency warning signs (dyspnea or complete anorexia) were reported by 39.1% of participants with anosmia (n=127,586) versus 22.7% of participants without anosmia (n=1,597,289; *P*<.001; Table 1). Anosmia and fever and/or cough were correlated with COVID-19–related hospitalizations (Spearman correlation coefficients=0.87 and 0.82, respectively; *P*<.001 for both; Figure 2).

**Table 1:** Users characteristics.

Characteristic		Value (n=2,477,174^a^)
Age (years), average (range), median		39.12 (15-99), 37
Sex		—^b^
**Body mass index (kg/m^2^)**		
	≥30	436,609 (17.6)
	<30	2,040,555 (82.4)
**Comorbidities**		
	Cardiovascular disease / uncontrolled hypertension	401,888 (16.2)
	Diabetes	78,354 (3.2)
	Malignancy	72,577 (2.9)
	Pulmonary disease	243,247 (9.8)
	Chronic kidney disease	11,333 (0.5)
	Chronic liver disease	36,677 (1.5)
	Pregnancy	40,766 (1.6)
	Immunodepression	135,624 (5.5)
	Immunosuppressive ongoing therapy	70,927 (2.9)
**Reported symptoms**		
	Fever (body temperature greater >37.7°C)	828,952 (33.5)
	Cough	1,515,557 (61.2)
	Dyspnea	658,442 (26.6)
	Asthenia	1,155,297 (46.6)
	Complete anorexia	103,122 (4.2)
	Sore throat or muscle aches	1,837,286 (74.1)
	Diarrhea	497,665 (20.1)
	Anosmia or dysgeusia^c^	325,910 (17.1)
**Patient triage after questionnaire completion^d^**		
	No symptoms	1,322,361 (34.8)
	Self-monitoring	858,878 (22.6)
	General practitioner / phone call	1,033,922 (27.2)

^a^Symptomatic patient (reported at least one symptom).

^b^Sex was not asked to protect the identity of the user.

^c^Anosmia was reported from March 21, 2020; the number of symptomatic patients during this period was 1,903,741.

^d^Denominator is N=3,799,535 (all respondents; symptomatic or not).

## Discussion

This study suggests that self-reported symptoms of COVID-19 are correlated with COVID-19–related hospitalizations and that anosmia may be strongly associated with COVID-19. This could be explained by a greater specificity compared to other reported symptoms that could result from other respiratory viruses [7,8]. Limitations include lack of medical assessment and virological confirmation of COVID-19 and comparison of application-retrieved data with a distinct set of data from the Ministry of Health reports on hospitalizations. Self-checking application data could be a relevant tool to monitor the dynamics of an outbreak and thus can be a real-time health system response to the epidemic.

## Abbreviations

COVID-19: coronavirus disease
GP: general practitioner
